# Genomic Characterization of Multidrug-Resistant *Salmonella* Serovars Derby and Rissen From the Pig Value Chain in Vietnam

**DOI:** 10.3389/fvets.2021.705044

**Published:** 2021-08-27

**Authors:** Belén González-Santamarina, Silvia García-Soto, Sinh Dang-Xuan, Mostafa Y. Abdel-Glil, Diana Meemken, Reinhard Fries, Herbert Tomaso

**Affiliations:** ^1^Institute of Bacterial Infections and Zoonoses, Friedrich-Loeffler-Institut, Jena, Germany; ^2^Institute of Institute of Molecular Pathogenesis, Friedrich-Loeffler-Institut, Jena, Germany; ^3^International Livestock Research Institute, Hanoi, Vietnam; ^4^Center for Public Health and Ecosystem Research, Hanoi University of Public Health, Hanoi, Vietnam; ^5^Institute of Food Safety and Food Hygiene, Section Meat Hygiene, Freie Universität Berlin, Berlin, Germany

**Keywords:** *Salmonella Rissen*, IncHI, multidrug resistance, pig value chain, Vietnam, *Salmonella Derby*

## Abstract

Nontyphoidal *Salmonella* (NTS) is the most reported cause of bacterial foodborne zoonoses in Vietnam, and contaminated pork is one of the main sources of human infection. In recent years, the prevalence of NTS carrying multiple antimicrobial resistance genes (ARGs) have been increased. The genomic characterization along the pig value chain and the identification of ARGs and plasmids have the potential to improve food safety by understanding the dissemination of ARGs from the farm to the table. We report an analysis of 13 *S*. Derby and 10 *S*. Rissen isolates, collected in 2013 at different stages in Vietnamese slaughterhouses and markets. VITEK 2 Compact System was used to characterize the phenotypical antimicrobial resistance of the isolates. In addition, whole-genome sequencing (WGS) was used to detect ARGs and plasmids conferring multidrug resistance. Whole genome single nucleotide polymorphism typing was used to determine the genetic diversity of the strains and the spread of ARGs along the pig value chain. Altogether, 86.9% (20/23) of the samples were resistant to at least one antibiotic. Resistance to ampicillin was most frequently detected (73.9%), followed by piperacillin and moxifloxacin (both 69.6%). At least one ARG was found in all strains, and 69.6% (16/23) were multidrug-resistant (MDR). The observed phenotype and genotype of antimicrobial resistance were not always concordant. Plasmid replicons were found in almost all strains [95.6% (22/23)], and the phylogenetic analysis detected nine clusters (*S*. Derby, *n* = 5; *S*. Rissen, *n* = 4). ARGs and plasmid content were almost identical within clusters. We found six MDR IncHI1s with identical plasmid sequence type in strains of different genetic clusters at the slaughterhouse and the market. In conclusion, high rates of multidrug resistance were observed in *Salmonella* strains from Vietnam in 2013. Genomic analysis revealed many resistance genes and plasmids, which have the potential to spread along the pig value chain from the slaughterhouse to the market. This study pointed out that bioinformatics analyses of WGS data are essential to detect, trace back, and control the MDR strains along the pig value chain. Further studies are necessary to assess the more recent MDR *Salmonella* strains spreading in Vietnam.

## Introduction

Nontyphoidal *Salmonella* (NTS) are among the most important foodborne pathogens worldwide (1). NTS infections were reported as the major cause of diarrhea in Vietnamese children and severe invasive infections in adults (2). NTS can be host generalists, infecting a broad range of vertebrates, including pigs (3). Pork is the most frequently consumed meat in Vietnam, but pigs commonly harbor this pathogen without showing clinical signs (4). The overall prevalence of *Salmonella* in pigs in Vietnam was 69.7% (5), and it varies at the different steps of the pig value chain. The prevalence in pig pen floors is between 27.7% (6) and 36.1% (4), and in slaughterhouses, pig carcasses are frequently contaminated (38.9 to 95.7%) (4, 7). Another relevant stage in the pig value chain are the markets. In Vietnam, fresh meat is usually bought on a traditional market, where it is displayed on tables or stands at ambient temperature (8). On pork markets, the prevalence of *Salmonella* in cut pork varied between 28.6 and 44.4% (4).

In Vietnam and in other Asian countries, *Salmonella* Rissen (*S*. Rissen) is the most prevalent serovar in pigs and in human patients with diarrhea (8). This serovar is also the most detected in pork, beef (5), and shrimp batches (2). Another important serovar is *S*. Derby, detected in pig lymph nodes with a prevalence of 50% (9). It was also detected in Vietnamese pig farms, pig carcasses in slaughterhouses, and pork in the market (4).

Antimicrobial resistance (AMR) transferred by bacteria is one of the greatest threats to both human and animal health (10). Humans can acquire bacterial infections encoding AMR genes (ARGs) through the consumption or handling of contaminated food (2). Multidrug-resistant bacterial isolates exhibit resistance to at least one agent in three or more antimicrobial families (11). In studies about the food distribution system in Vietnam, multidrug-resistant (MDR) *Salmonella* strains were widely disseminated in retail meat and seafood (13) #185; (12) #173; (5) #174. In retail meat, 52 to 92.8% of the samples were resistant to at least one antibiotic, and between 36 and 64.3% carried MDR *Salmonella* (12, 13). In seafood, 50 and 37.5% of fish and shrimps' samples were resistant, respectively (5).

Resistance against tetracycline, trimethoprim–sulfamethoxazole, chloramphenicol, and ampicillin is the most commonly reported in *Salmonella* isolates collected from pork, other retail meat, and food products in Vietnam (5, 9, 12). Furthermore, in Vietnam, Extended Spectrum Beta-Lactamase (ESBL)- and AmpC-producing *Salmonella* isolates have been detected in food (5) and *mcr-1* in poultry meat (14). This high MDR prevalence could be related to the use of large amounts of antimicrobials to treat infections, as well as to increase the productivity of animal farming (8). Horizontal gene transfer plays an important role in the spread of AMR in bacterial populations (15). The most common vehicles linked to the dissemination of ARGs are plasmids with a wide range of sizes (15, 16). In *Salmonella*, the most common conjugative plasmids are those that belong to incompatibility groups (Inc) IncI, IncFI, IncN, and IncH (16), which carry a great variety of ARGs (17).

During the last years, whole-genome sequencing (WGS) has been shown to be useful for genetic characterization of *Salmonella* isolates including serovar prediction (18). To our knowledge, WGS is not yet established in Vietnam for NTS analysis, and most of the studies still rely on conventional microbiological methods (19).

In this study, we used WGS and bioinformatics analysis to decipher the genetic traits (ARGs and plasmid replicons) of *S*. Derby and *S*. Rissen isolates collected in 2013 in Vietnam at different stages of the pig value chain (pig slaughterhouses and pork markets). We performed phenotypic characterization and AMR testing, as well as a phylogenetic analysis using a distance matrix based on single-nucleotide polymorphisms (SNPs). We also predicted AMR based on WGS data. Plasmids carrying ARGs were traced for potential dissemination through the pig value chain. The aim of this study was to show that WGS could potentially help to detect and to control the dissemination of ARGs from the farm to the table in Vietnam.

## Materials and Methods

### Bacterial Strains, Isolation, and Identification

In this study, 23 *Salmonella* isolates (13 *S*. Derby and 10 *S*. Rissen) were analyzed. They were collected from diverse positions in pig slaughterhouses and pork markets in Hung Yen province in Vietnam in 2013 (Table 1). These strains were provided by Univ.-Prof. i. R. Dr. Reinhard Fries from the Institute of Food Safety and Food Hygiene Working Group Meat Hygiene of Freie Universität Berlin (FU). They were isolated and serotyped following the DIN EN ISO 6579-1:2017-07 in 2013 and stored on glycerol or cryotubes at −20°C. In July 2019, these strains were transferred from the Institute of Food Safety and Food Hygiene, Working Group Meat Hygiene, FU, to the Institute of Bacterial Infections and Zoonoses (IBIZ, Jena) under appropriate shipping conditions.

**Table 1:** Bioinformatics results of the 23 isolates of *Salmonella enterica* subsp. *enterica* serovar Derby and Rissen.

**Metadata**				**Antibiotic resistance genes**		**Plasmid**	
**Serovar**	**ST**	**ID strain**	**Sample**	***n***	**Gene names**	***n***	**Replicon name**
*S*. Derby	40	19CS0392	Slaughterhouse worker hands	12	*bla_*TEM*.1_, aadA1, aadA2, qnrS1, fosA7.3, dfrA12, sul2, sul3, tetA, tetM, cmlA1, floR*	0	0
*S*. Derby	40	19CS0393	Swab on pig carcass at slaughterhouse	12	*bla_*TEM*.1_, aadA1, aadA2, qnrS1, fosA7.3, dfrA12, sul2, sul3, tetA, tetM, cmlA1, floR*	1	Col440I_1
*S*. Derby	40	19CS0395	Pork at market	12	*bla_*TEM*.1_, aadA1, aadA2, qnrS1, fosA7.3, dfrA12, sul2, sul3, tetA, tetM, cmlA1, floR*,	1	Col440I_1
*S*. Derby	40	19CS0397	Skin on pig carcass	13	*bla_*TEM*.1_, bla_*LAP*2_, aac(3)-IId, aadA1, aadA2, qnrS1, fosA7.3, dfrA12, sul2, sul3, tetA, cmlA1, mefB*	2	ColRNAI_1, IncFIB.K._1_Kpn3
*S*. Derby	40	19CS0399	Slaughterhouse worker hands	13	*bla_*TEM*.1_, bla_*LAP*2_, aac(3)-IId, aadA1, aadA2, qnrS1, fosA7.3, dfrA12, sul2, sul3, tetA, cmlA1, mefB*	2	Col440II_1, IncFIB.K._1_Kpn3
*S*. Derby	40	19CS0400	Pork at market	7	*bla_*TEM*.1_, qnrS1, fosA7.3, sul2, tetA, tetM, floR*	5	IncHI1A_1, IncHI1B.R27._1_R27IncFIA.HI1._1_HI1, Col440I_1, ColRNAI_1
*S*. Derby	40	19CS0401	Pork at market	7	*bla_*TEM*.1_, qnrS1, fosA7.3, sul2, tetA, tetM, floR*	5	IncHI1A_1, IncHI1B.R27._1_R27IncFIA.HI1._1_HI1, Col440I_1, ColRNAI_1
*S*. Derby	40	19CS0402	Pork at market	11	*bla_*TEM*.1_, aadA1, aadA2, qnrS1, fosA7.3, dfrA12, sul2, sul3, tetA, tetM, cmlA1, floR*	5	IncHI1A_1, IncHI1B.R27._1_R27IncFIA.HI1._1_HI1, Col440I_1, ColRNAI_1
*S*. Derby	40	19CS0403	Pork at market	7	*bla_*TEM*.1_, fosA7.3, qnrS1, sul2, floR, tetA, tetM*	5	IncHI1A_1, IncHI1B.R27._1_R27IncFIA.HI1._1_HI1, Col440I_1, ColRNAI_1
*S*. Derby	40	19CS0404	Pork at market	8	*aadA1, aadA2, qnrS1, fosA7.3, dfrA12, sul3, tetM, cmlA1*	2	IncHI1A_1, IncHI1B.R27._1_R27
*S*. Derby	40	19CS0418	Cutting board at pork shop	13	*bla_*TEM*.1_, bla_*LAP*2_, aac(3)-IId, aadA1, aadA2, qnrS1, fosA7.3, dfrA12, sul2, sul3, tetA, cmlA1, mefB*	2	IncFIB.K._1_Kpn3, Col440II_1
*S*. Derby	40	19CS0419	Pork at market	12	*bla_*TEM*.1_, aadA1, aadA2, qnrS1, fosA7.3, dfrA12, sul2, sul3, tetA, tetM, cmlA1, floR*	1	Col440I_1
*S*. Derby	40	19CS0424	Slaughterhouse worker hands	12	*bla_*TEM*.1_, aadA1, aadA2, fosA7.3, qnrS1, dfrA12, sul2, sul3, tetA, tetM, cmlA1, floR*	1	Col440I_1
*S*. Rissen	469	19CS0394	Pork at market	1	*tetA*	2	Col440II_1, ColRNAI_1
*S*. Rissen	469	19CS0396	Swab on pig carcass at slaughterhouse	1	*tetA*	2	Col440II_1, ColRNAI_1
*S*. Rissen	469	19CS0398	Splitting place at pig slaughterhouse	1	*tetA*	5	Col440II_1, ColRNAI_1
*S*. Rissen	469	19CS0416	Skin in carcass	8	*bla_*TEM*.1_, aac(3)-IId, aadA2, mcr-1, tetA, tetM, floR, lnuF*	7	IncFIB.AP001918._1, IncFIC.FII._1, IncI2_1_Delta, Col.BS512._1, Col156_1, Col440II_1, ColRNAI_1
*S*. Rissen	469	19CS0417	Splitting place at pig slaughterhouse	10	*bla_*TEM*.1_, aadA1, aadA2, qnrS1, dfrA12, sul2, sul3, tetM, cmlA1, floR*	4	IncHI1A_1, IncHI1B.R27._1_R27, Col440II_1, ColRNAI_1
*S*. Rissen	469	19CS0420	Pork at market	1	*tetA*	2	Col440II_1, ColRNAI_1
*S*. Rissen	469	19CS0421	Pork at market	1	*tetA*	2	Col440II_1, ColRNAI_1
*S*. Rissen	469	19CS0422	Pork at market	8	*bla_*TEM*.1_, aadA1, aadA2, dfrA12, sul1, sul3, tetA, cmlA1*	3	IncY_1, Col440II_1, ColRNAI_1
*S*. Rissen	69	19CS0423	Swab on pig carcass at slaughterhouse	1	*tetA*	2	Col440II_1, ColRNAI_1
*S*. Rissen	469	19CS0425	Slaughterhouse worker hands	1	*tetA*	2	Col440II_1, ColRNAI_1

Isolates were resuspended in 3 mL of brain heart infusion broth (Mast Diagnostica GmbH, Reinfeld, Germany) and incubated between 4 and 18 h at 37°C. They were cultivated on RAMBACH^®^ Agar (Merck KGaA, Darmstadt, Germany) for 24 ± 3 h at 37°C. The cultures were not contaminated, and the colonies had a characteristic red–pink color with a bright edge.

### Antimicrobial Susceptibility Testing

The isolates were recultivated on Columbia blood plates for 24 h at 37°C. Antimicrobial susceptibility test (AST) of the strain was assessed by determining the minimum inhibitory concentration (MIC) using the VITEK 2 Compact System (bioMérieux, Marcy-l'Étoile, France). VITEK cards AST-N195 and AST-N248 were employed to determine MIC values (mg/L) according to the European Committee on Antimicrobial Susceptibility Testing (EUCAST) guidelines (20). Twenty-four different antibiotics were evaluated: ampicillin, amoxicillin–clavulanic acid, piperacillin, piperacillin–tazobactam, cefalexin, cefuroxime, cefuroxime–axetil, cefotaxime, ceftazidime, cefepime, aztreonam, ertapenem, imipenem, meropenem, amikacin, gentamicin, tobramycin, ciprofloxacin, tigecycline, fosfomycin, colistin, trimethoprim, and trimethoprim–sulfamethoxazole. VITEK 2 Compact System (bioMérieux, Marcy-l'Étoile, France) also detected the EUCAST epidemiological cutoff values (ECOFFs). Here the 24 antibiotics are enclosed in seven antimicrobial families: β-lactam antibiotics, aminoglycosides, quinolone, fosfomycin, trimethoprim/sulfonamide, polypeptide, and tetracycline. MIC values (mg/L) of fosfomycin in *S*. Derby isolates were also tested with Micronaut AST-system plate M/E1-055-040 (MERLIN Diagnostika, Bornheim-Hersel, Germany) following the manufacturer's instructions and according to EUCAST guidelines. Turbidity reading and interpretation were done manually.

### Whole-Genome Sequencing

For sequencing, the 23 *Salmonella* isolates were grown overnight at 37°C in 3 mL of Luria-Bertani broth (Mast Diagnostica GmbH, Reinfeld, Germany). DNA extraction was performed using the DNeasy blood and tissue kit (QIAGEN GmbH, Hilden, Germany) following the manufacturer's instructions for Gram-negative bacteria. DNA sequencing libraries were constructed using the Nextera XT Preparation Kit (Illumina Inc., San Diego, CA) following the manufacturer's instructions. Paired-end sequencing was performed on an Illumina MiSeq platform (Illumina Inc.) using a 300-cycle MiSeq reagent kit.

One strain (19CS0402) was additionally sequenced using the MinION platform to analyze the complete genome sequence and the plasmid structure. High-molecular-weight DNA was extracted using Genomic-tip 100/G and genomic DNA buffer kit (QIAGEN GmbH). The sequencing library was prepared using the Oxford Nanopore Technologies 1D Ligation Sequencing Kit (SQK-LSK109) with the Native Barcoding Expansion Kit (EXP-NBD104) as recommended by the manufacturer.

### Bioinformatics Analysis

We performed bioinformatics analysis of the strains using an in-house Linux-based bioinformatics pipeline called WGSBAC v.2.0 (available in its last version https://gitlab.com/FLI_Bioinfo/WGSBAC/). WGSBAC takes as input Illumina raw paired-end reads, and the quality of the sequences is checked using FastQC v0.11.7 (http://www.bioinformatics.babraham.ac.uk/projects/fastqc/). The coverage of the raw reads is estimated theoretically as the total number of sequenced bases/reference genome size using an adapted script (https://github.com/raymondkiu/fastq-info/blob/master/fastq_info_3.sh). Reads are *de novo* assembled using Shovill v.1.0.4 (21) and evaluated by QUAST v.5.0.2 (22) in standard settings. Annotation is performed with Prokka v.1.14.5 (23), and to identify contamination, the pipeline uses Kraken 2 v.1.1 (24) and Kraken2DB to classify both reads and contigs. Acquired ARGs are detected using the software AMRFinderPlus v.3.6.10 (25). Additionally, ABRicate v.0.8.10 (available at https://github.com/tseemann/abricate) with the databases CARD v.3.0.8 (26) and ResFinder v.3.2 (27) is used for the detection of ARGs. For the identification of plasmid replicon, ABRicate uses PlasmidFinder v.2.0.1 (28). For *in silico* serotyping, the WGSBAC pipeline relies on SISTR v.1.0.2 (29). For genotyping, WGSBAC uses classic multilocus sequence typing (MLST) using the software mlst v.2.16.1 on assembled genomes. For plasmid typing, we used the plasmid MLST (pMLST) 2.0 external server v.1.3 with the plasmid PubMLST database (https://pubmlst.org/plasmid/).

Furthermore, WGSBAC uses Snippy v.4.3.6 for core-genome SNP (cgSNP) detection. FastTree v.2.1.10 (30) uses the SNP distance matrix obtained by Snippy v.2.1.10 (30) to build a cgSNP-based phylogenetic tree. For visualization of the phylogenetic trees, we employed the online tool iTOL v.5.1.0 (31).

The IncHI1 plasmid–positive strain 19CS0402 (p19CS0402-IncHI1) was sequenced using an Illumina MiSeq platform (Illumina Inc.), as well as with a MinION sequencer (Oxford Nanopore Technologies, Oxford, United Kingdom) to close sequences of the chromosome and the plasmid. The raw FAST5 files from the MinION were processed using the Guppy toolkit v.3.4.1 (Oxford Nanopore Technologies). The Guppy command *guppy_basecaller* was used for base-calling, and *guppy_barcoder* was used for demultiplexing. *De novo* assembly for long sequencing reads was performed using Flye v.2.6 (32). Assembly polishing was performed first using the long reads in four rounds by Racon v.1.4.3 (33) and one final round with Medaka v.0.10.0. Next, Illumina reads were trimmed using Trimmomatic v.0.39, and Pilon v.1.23 (34) was used to correct the assembled data obtained with the MinION with the trimmed Illumina reads using standard settings.

**Figure 1:**
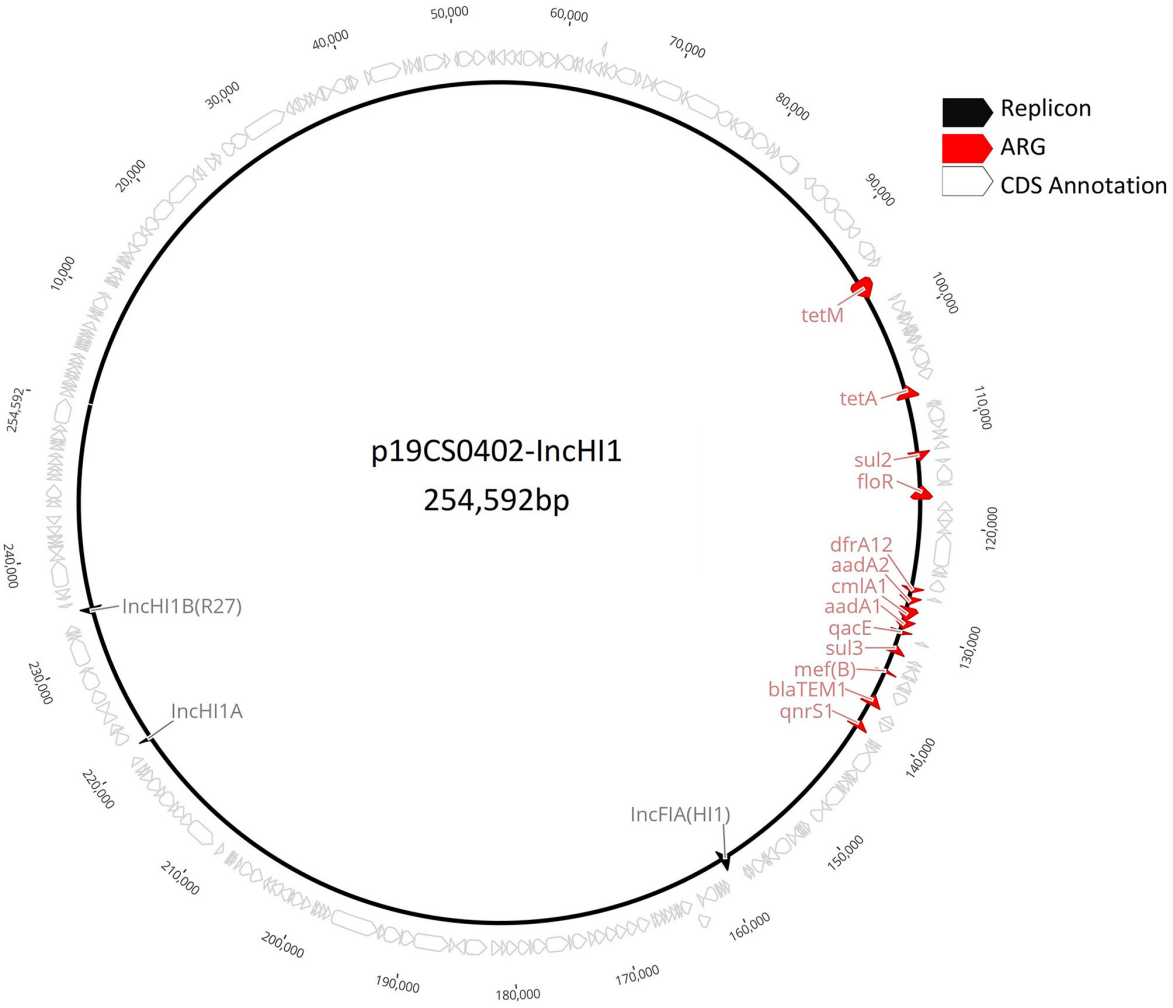
Complete plasmid sequence of p19CS0402-IncHI1 from the Vietnamese strain 19CS402.

To compare the IncHI1 plasmids of the same plasmid ST replicon, the whole p19CS0402-IncHI1 (Figure 1) sequence was mapped against the Illumina draft sequences carrying the same replicon. The multiple alignment was performed by the algorithm Mauve (35) within the external software Geneious Prime v.11.1.5 (Biomatters, Ltd., Auckland, New Zealand). Annotation was performed with Prokka v.1.14.5 (23) (Supplementary Table 1).

## Results

### Serovar Prediction and Genotyping

The *in silico* serotyping tool SISTR (v1. 0.2) confirmed all serotyped isolates as *S*. Derby (*n* = 13) with antigen formula 1,4,5,[12]:f,g:-, according to the White–Kauffmann–Le Minor scheme (36). In addition, all the isolates serotyped as *S*. Rissen (*n* = 10) were confirmed as serovar Rissen with the antigenic formula 6,7,14:f,g:-. Classic MLST revealed sequence type (ST) 40 [*aroC* (19), *dnaN* (20), *hemD* (3), *hisD* (20), *purE*(5), *sucA* (23), and *thrA* (23)] for all *S*. Derby strains and ST469 for all *S*. Rissen isolates [*aroC* (92), *dnaN* (107), *hemD* (79), *hisD* (156), *purE* (64), *sucA* (151), and *thrA* (87)] (Table 1).

### Antimicrobial Susceptibility Testing

The 23 isolates were successfully processed with the VITEK 2 Compact System (bioMérieux, Marcy-l'Étoile, France) to determine the MIC (mg/L) values of 24 different antibiotics. Eighty-seven percent (20/23) of the samples were resistant to at least one antibiotic (Table 2). The most frequently detected resistance was against ampicillin (73.9%) and piperacillin and moxifloxacin (both 69.6%), followed by trimethoprim and trimethoprim with sulfamethoxazole (both 56.5%, Table 2). Resistance to cefuroxime–acetyl, gentamicin, tobramycin, and tigecycline was found in 17.4% isolates, followed by ciprofloxacin (13%) and colistin (4.3%, Table 2). The antibiotics categorized as “susceptible, increased exposure” (new intermediate nomenclature by EUCAST) (37) were amoxicillin–clavulanic acid (65.2%), tigecycline (21.7%), and piperacillin–tazobactam (4.3%). All other tested antibiotics were classified as susceptible (standard dosing regimen) (Table 2; Supplementary Table 2).

**Table 2:** AST results of the 23 *Salmonella* isolates.

**Antibiotic class**	**Antibiotic**	**Resistant**		**Susceptible, increased exposure (I)**		**Susceptible**	
		***n***	**%**	***n***	**%**	***n***	**%**
β-Lactams	AMP	17	73.9%	–	–	6	26.1%
	AMC	–	–	15	**65.2%**	8	34.8%
	PIP	16	69.6%	–	–	7	30.4%
	PIT	–	–	1	4.3%	22	95.7%
	CTN	–	–	–	–	23	100%
	CURA	4	17.4%	–	–	19	82.6%
	CTA	–	–	–	–	23	100%
	CTZ	–	–	–	–	23	100%
	CEP	–	–	–	–	23	100%
	AZT	–	–	–	–	23	100%
Carbapenem	ERT	–	–	–	–	23	100%
	IMI	–	–	–	–	23	100%
	MER	–	–	–	–	23	100%
Aminoglycosides	AMI	–	–	–	–	23	100%
	GEN	4	17.4%	–	–	19	82.6%
	TOB	4	17.4%	–	–	19	82.6%
Quinolones	CIP	3	13.0%	–	–	20	87.0%
	MOX	16	69.6%	–	–	9	39.1%
Fosfomycin	FOS	–	–	–	–	23	100%
Trimethoprim/sulfamethoxazole	TRI	13	56.5%	–	–	10	43.5%
	T/S	13	56.5%	–	–	10	43.5%
Polymyxins	COL	1	4.3%	–	–	22	95.7%
Tetracyclines	TIG	4	17.4%	5	21.7%	14	60.9%

*AMP, ampicillin; AMC, amoxicillin/clavulanic acid; PIP, piperacillin; PIT, piperacillin–tazobactam; CLE, cefalexin; CUR, cefuroxime; CURA, cefuroxime axetil; CTA, cefotaxime; CTZ, ceftazidime; CEP, cefepime; AZT, aztreonam; ERT, ertapenem; IMI, imipenem; MER, meropenem; AMI, amikacin; GEN, gentamicin; TOB, tobramycin; CIP, ciprofloxacin; MOX, moxifloxacin; TRI, trimethoprim; TRS, trimethoprim–sulfamethoxazole; TIG, tigecycline; FOS, fosfomycin; COL, colistin*.

### ARG Detection

ARGs were found in all isolates (Table 1). They belonged to 11 antibiotics classes (Table 3): tetracycline (100%; *tetA* and/or *tetM*), β-lactam antibiotics (65.2%; *bla*_*TEM*.1_and/or *bla*_*LAP*_), chloramphenicol (69.6%; *cmlA1* and/or *floR*), sulfonamide (65.2%; *sul1, sul2*, and/or *sul3*), quinolone (60.9%; *qnrS1*), aminoglycoside (56.5%; *aadA1, aadA2*, and/or *aac(3)-IId*), fosfomycin (56.5%; *fosA7.3*), trimethoprim (52.5%; *dfrA12*) macrolide [13%; *mef(B*)], polypeptide (4.3%, *mcr-1*), and lincosamide (4.3%; *lnuF*) (Table 3). All *S*. Derby isolates carried ARGs to at least six different antibiotics classes (β-lactam antibiotics, quinolone, fosfomycin, trimethoprim–sulfonamide, tetracycline, and chloramphenicol). However, the only ARG present in all *S*. Rissen was against tetracyclines (Table 1).

**Table 3:** Resistance genes of the 23 isolates of *Salmonella enterica* subsp. *enterica* serovar Derby and Rissen.

**Resistance antibiotic families**	**Total %**	**Genes**	**%**
Tetracycline	100	*tetA*	91.3
		*tetM*	52.2
β-Lactams	65.2	*bla_*TEM*.1_*	65.2
		*bla_*LAP*_*	13
Chloramphenicol	69.6	*cmlA1*	52.2
		*floR*	47.8
Sulfonamide	65.2	*sul1*	4.3
		*sul2*	56.5
		*sul3*	52.2
Quinolone	60.9	*qnrS1*	60.9
Aminoglycoside	56.2	*aadA1*	52.2
		*aadA2*	56.5
		*aac(3)-IId*	17.4
Fosfomycin	56.5	*fosA7.3*	56.5
Trimethoprim	52.2	*dfrA12*	52.2
Macrolide	13	*mef(B)*	13
Polypeptide	4.3	*mcr-1*	4.3
Lincosamide	4.3	*lnuF*	4.3

### Comparison of Phenotypic and Genotypic Antimicrobial Profiles

The correlation between the phenotypic resistance and the ARGs was compared. Discrepant results between the detected phenotype and the genotype were found in 20 isolates. The most discordant results were found in aminoglycosides, tetracycline, and fosfomycin (Table 4). In the case of aminoglycosides, 19 strains were detected as wild type, but nine of them carried two or three ARGs against aminoglycosides (Table 4). In addition, all the isolates had at least one ARG to tetracycline; nevertheless, 14 strains identified as wild type carried one or two genes against this antibiotic. Besides, all strains were identified as fosfomycin wild type and susceptible in the VITEK 2 Compact System (Table 4) and Micronaut AST-system, respectively (Supplementary Table 2). However, all the *S*. Derby strains carried *fosA7.3* gene. *S*. Derby strains 19CS0419 and 19CS0424 (Table 4) carried different ARGs, but they were identified as wild type. In contrast, four *S*. Rissen isolates (19CS0394, 19CS0396, 19CS0398, and 19CS0425; Table 4) were classified as non-sensitive to β-lactam antibiotics, trimethoprim–sulfonamide and quinolones, but no resistance genes were detected.

**Table 4:** Comparison of antibiotic resistance phenotype and genotype of the 15 *Salmonella* Derby samples.

**Metadata**			**β-Lactam antibiotics**		**Aminoglycoside**		**Quinolone**		**Fosfomycin**		**Trimethoprim/sulfonamide**		**Polypeptide**		**Tetracycline**	
**Serotype**	**ID strain**	**Sample**	**P**	**G**	**P**	**G**	**P**	**G**	**P**	**G**	**P**	**G**	**P**	**G**	**P**	**G**
*S*. Derby	19CS0392	Slaughterhouse worker hands	AP	*bla_*TEM*.1_*	W	*aadA1, aadA2*	PR	qnrS1	W	fosA7.3	R	*sul2, sul3, dfrA12*	W	—	W	*tetA, tetM*
*S*. Derby	19CS0393	Swab on pig carcass at slaughterhouse	AP	*bla_*TEM*.1_*	W	*aadA1, aadA2*	PR	qnrS1	W	fosA7.3	R	*sul2, sul3, dfrA12*	W	—	W	*tetA, tetM*
*S*. Derby	19CS0395	Pork at market	AP	*bla_*TEM*.1_*	W	*aadA1, aadA2*	PR	qnrS1	W	fosA7.3	R	*sul2, sul3, dfrA12*	W	—	W	*tetA, tetM*
*S*. Derby	19CS0397	Skin on pig carcass	AP	*bla_*TEM*.1_, bla_*LAP*2_*	R	*aadA1, aadA2, aac(3)-IId*	R	qnrS1	W	fosA7.3	R	*sul2, sul3, dfrA12*	W	—	R	*tetA*
*S*. Derby	19CS0399	Slaughterhouse worker hands	AP	*bla_*TEM*.1_, bla_*LAP*2_*	R	*aadA1, aadA2, ,aac(3)-IId*,	R	qnrS1	W	fosA7.3	R	*sul2, sul3, dfrA12*	W	—	R	*tetA*
*S*. Derby	19CS0400	Pork at market	AP	*bla_*TEM*.1_*	W	—	PR	qnrS1	W	fosA7.3	W	*sul2*	W	—	W	*tetA, tetM*
*S*. Derby	19CS0401	Pork at market	AP	*bla_*TEM*.1_*	W	—	PR	qnrS1	W	fosA7.3	W	*sul2*	W	—	W	*tetA, tetM*
*S*. Derby	19CS0402	Pork at market	AP	*bla_*TEM*.1_*	W	*aadA1, aadA2*	PR	qnrS1	W	fosA7.3	R	*sul2, sul3, dfrA12*	W	—	W	*tetA, tetM*
*S*. Derby	19CS0403	Pork at market	AP	*bla_*TEM*.1_*	W	—	PR	qnrS1	W	fosA7.3	W	*sul2*	W	—	W	*tetA, tetM*
*S*. Derby	19CS0404	Pork at market	AP	-	W	*aadA1, aadA2*	R	qnrS1	W	fosA7.3	R	*sul3, dfrA12*	W	—	R	*tetM*
*S*. Derby	19CS0418	Cutting board at pork shop	W	*bla_*TEM*.1_, bla_*LAP*2_*	W	*aadA1, aadA2, ,aac(3)-IId*,	W	qnrS1	W	fosA7.3	W	*sul2, sul3, dfrA12*	W	—	W	*tetA*
*S*. Derby	19CS0419	Pork at market	W	*bla_*TEM*.1_*	W	*aadA1, aadA2*	W	qnrS1	W	fosA7.3	W	*sul2, sul3, dfrA12*	W	—	W	*tetA, tetM*
*S*. Derby	19CS0424	Slaughterhouse worker hands	W	*bla_*TEM*.1_*	W	*aadA1, aadA2*	W	qnrS1	W	fosA7.3	W	*sul2, sul3, dfrA12*	W	—	R	*tetA, tetM*
*S*. Rissen	19CS0394	Pork at market	AP	—	W	—	PR	—	W	—	R	—	W	—	W	*tetA*
*S*. Rissen	19CS0396	Swab on pig carcass at slaughterhouse	AP	—	W	—	PR	—	W	—	R	—	W	—	W	*tetA*
*S*. Rissen	19CS0398	Splitting place at pig slaughterhouse	AP	—	R	—	R	—	W	—	R	—	W	—	R	*tetA*
*S*. Rissen	19CS0416	Skin in carcass	AP	*bla_*TEM*.1_*	R	*aadA2, ,aac(3)-IId,*	W	—	W	—	W	—	R	*mcr-1.1*	R	*tetA, tetM*
*S*. Rissen	19CS0417	Splitting place at pig slaughterhouse	AP	*bla_*TEM*.1_*	W	*aadA1, aadA2*	PR	qnrS1	W	—	R	*sul2, sul3, dfrA12*	W	—	W	*tetM*
*S*. Rissen	19CS0420	Pork at market	W	—	W	—	W	—	W	—	W	—	W	—	R	*tetA*
*S*. Rissen	19CS0421	Pork at market	W	—	W	—	W	—	W	—	W	—	W	—	W	*tetA*
*S*. Rissen	19CS0422	Pork at market	AP	*bla_*TEM*.1_*	W	*aadA1, aadA2*	W	—	W	—	R	*sul1, sul3, dfrA12*	W	—	R	*tetA*
*S*. Rissen	19CS0423	Swab on pig carcass at slaughterhouse	W	—	W	—	W	—	W	—	W	—	W	—	R	*tetA*
*S*. Rissen	19CS0425	Slaughterhouse worker hands	AP	—	W	—	PR	—	W	—	R	—	W	—	W	*tetA*

*P, phenotype determinate by VITEK Compact 2; G, genotype determinate by ResFinder; AD, acquired penicillinase; W, wild type; R, resistance; PR, partial resistance; I, intermediate*.

### Phylogenetic Analysis

**Figure 2:**
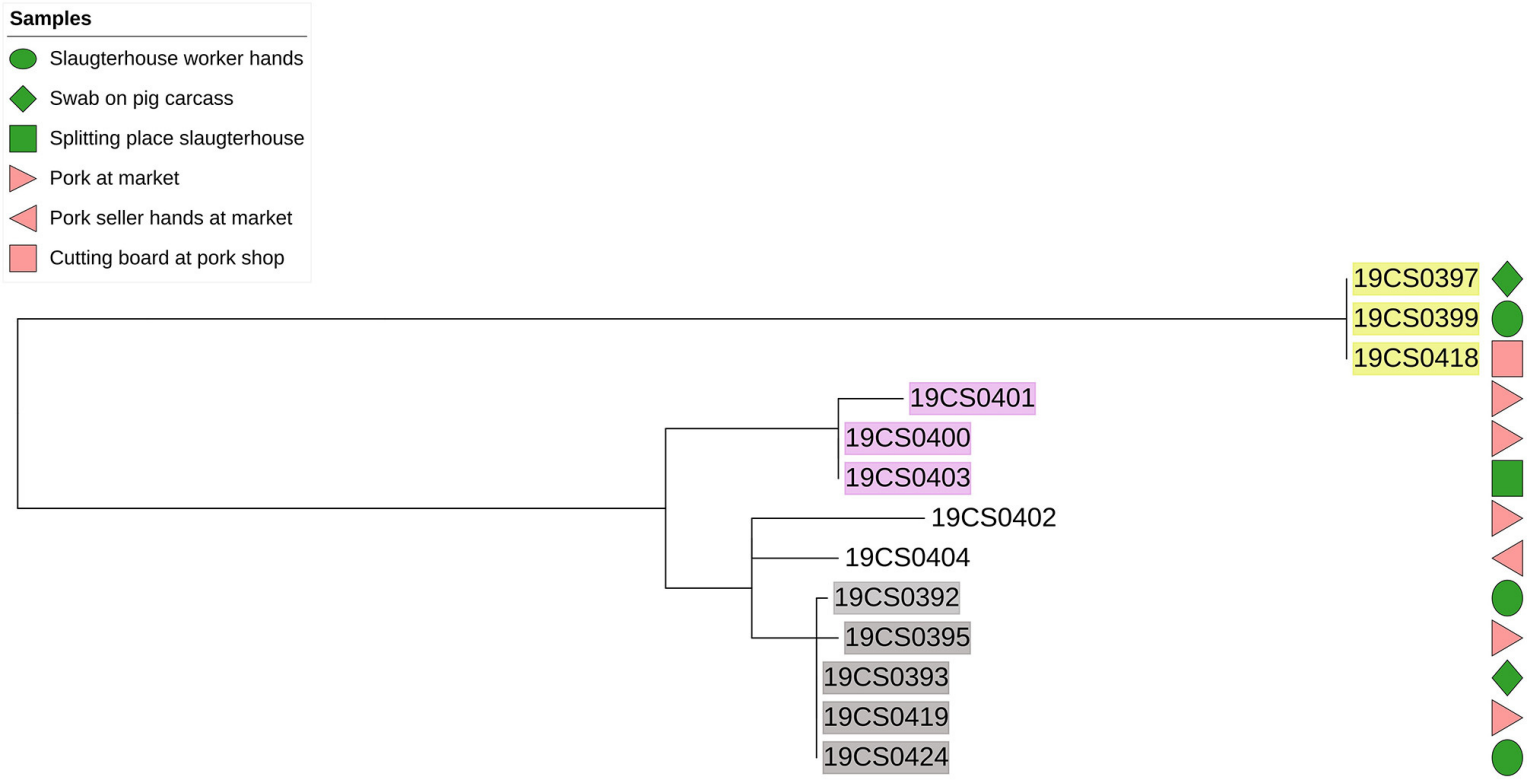
Phylogenetic SNP-based tree of *S*. Derby strains. Samples highlighted in different colors: clusters with more than one isolate. Green color figure: slaughterhouse samples, pink color figures: market samples.

The phylogenetic distance among *S*. Derby strains was on average 89 SNPs (0–207 SNPs; Supplementary Table 3). They were grouped in six different clusters (Figure 2). Three clusters carried more than one strain (highlighted in different colors in Figure 2). Strains within the same cluster had a distance between up to six SNPs (Supplementary Table 3). Additionally, two of these clusters (yellow and gray highlight, Figure 2) contained strains taken in the slaughterhouses and markets.

**Figure 3:**
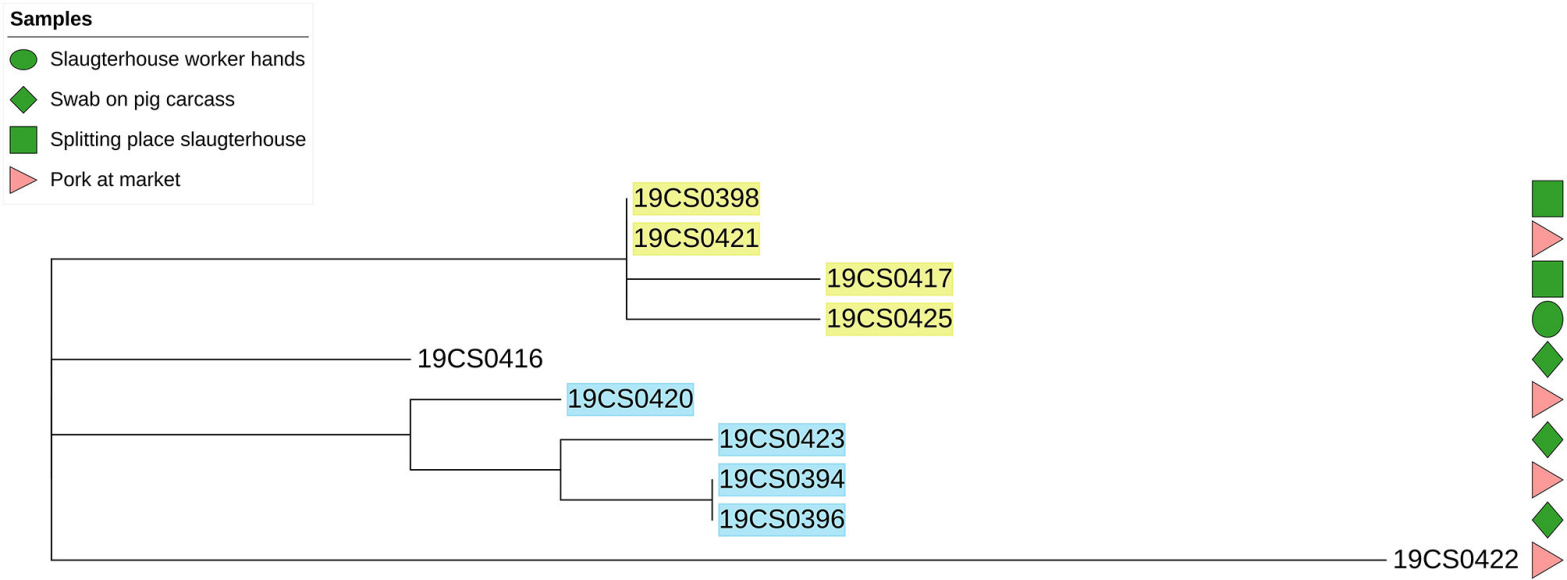
Phylogenetic SNP-based tree of *S*. Rissen strains. Samples highlighted in different colors: clusters with more than one isolate. Green color figures: slaughterhouse samples, red color figures: market samples.

The average of phylogenetic distance in *S*. Rissen strains was 32 SNPs (0–64 SNPs; Supplementary Table 3). They were grouped into four different clusters. Two of them contained strains taken in the slaughterhouse and the market (yellow and blue highlight, Figure 3). The distance among the *S*. Rissen strain clusters was up to six SNPs (Supplementary Table 3).

### Plasmid Replicon Identification

The 95.6% (22/23) of the strains carried at least one plasmid replicon (Table 1). The most frequently detected replicons belong to the Col-plasmids (Col440II_1, Col440I_1, ColRNAI_1, Col.BS512._1 and Col156_1). They were detected in 91.3% (21/23) of the samples. Moreover, incompatibility group (Inc) replicons IncFI (IncFIA, IncFIB, and IncFIC) were found in 34.7% (8/23) of the isolates and IncHI1 [IncHI1A and IncHI1B(R27)] in 26.1% (6/23). In addition, IncI and IncY were detected once in single isolate each (Table 1).

**Figure 4:**
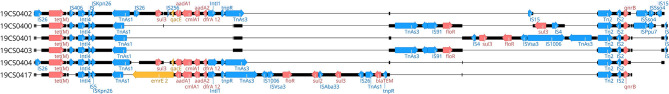
Plasmids IncHI alignment of region, where ARGs are located. Blue annotation: mobile genetic elements [e.g., transposons (Tn) and insertion sequences (IS)]. Red annotations: resistance genes. Yellow annotations: efflux pumps or antiseptic resistance genes.

pMLST analysis revealed six IncHI1 plasmid replicons with the identical (so far unknown) plasmid ST, which was closest to ST 16. The plasmid p19CS0402-IncHI1 carried also a IncFIA(HI1) replicon (Figure 1). The alignment of the six plasmids showed between 54.03 and 92.5% identity (Supplementary Table 4). The replicon IncFIA(HI1) was also detected in isolates 19CS0400, 19CS0401, and 19CS0403. Within the plasmids, differences in ARGs surrounded mobile genetic elements (MGEs) [insertion sites (IS) and transposons (Tn)] were found in a region of 81,503 bp (Figure 4). These plasmids carried between three and nine ARGs, and all sequences that contained *tetM* and *qnrS1* were surrounded by similar MGEs (Figure 4). Between these two genes, other ARGs were located near MGEs. The isolate 19CS402 carried *sul2, sul3, aad1, cmlA1*, and *aad2* (Figure 4). The isolate 19CS0417 contained additionally *floR, bla*_TEM_, and *emrE*, which is a multidrug resistance antibiotic efflux pump (Figure 4). Moreover, 19CS402 and 19CS404 carried the *qacE* gene, which confers resistance to antiseptics.

### Genetic Clusters and Their Occurrence in the Pig Value Chain

SNP-based phylogeny revealed 10 clusters (Table 5). Five of them carried more than one isolate (three *S*. Derby and two *S*. Rissen). They were collected in the pig slaughterhouse (splitting place, worker's hand, and/or swab on pig carcass) and market (pork at market and/or cutting board) (Table 5). Almost all isolates of the same cluster carried the same ARGs and plasmid replicons. The only exceptions were 19CS392 (*S*. Derby, cluster 1) and 19CS0417 (*S*. Rissen, cluster 1) (Table 5, indicated with an ^*^). In 19CS392, no Col440_1 plasmid was detected, and 19CS0417 carried more ARGs and plasmid replicons than the other strains in the same cluster (Table 5).

**Table 5:** Isolates organized in clusters with information about the collection place, resistance genes, and plasmid replicons.

**Serovar**	**Cluster**	**Slaughterhouse**			**Market**		**WGS results**	
		**Splitting place**	**Worker hands**	**Swab on pig carcass**	**Pork**	**Cutting board**	**Resistance genes**	**Plasmid's replicons**
Derby	1		19CS0392^*^ 19CS0424	19CS0393	19CS0395 19CS0419		*blaTEM.1, aadA1, aadA2, qnrS1, fosA7.3, dfrA12, sul2, sul3, tetA, tetM, cmlA1, floR*	^*^/Col440I_1
	2		19CS0399	19CS0397		19CS0418	*blaTEM.1, blaLAP2, aac(3)-IId, aadA1, aadA2, qnrS1, fosA7.3, dfrA12, sul2, sul3, tetA, cmlA1, mefB*	ColRNAI_1, IncFIB.K
	3				19CS0402		*blaTEM.1, aadA1, aadA2, qnrS1, fosA7.3, dfrA12, sul2, sul3, tetA, tetM, cmlA1, floR*	IncHI1A_1, IncHI1B.R27._1_R27, IncFIA.HI1._1_HI1, , Col440I_1, ColRNAI_1
	4				19CS0404		*aadA1, aadA2, qnrS1, fosA7.3, dfrA12, sul3, tetM, cmlA1*	IncHI1A_1, IncHI1B.R27._1_R27
	5	19CS0403			19CS0400 19CS0401		*blaTEM.1, qnrS1, fosA7.3, sul2, tetA, tetM, floR*	IncHI1A_1, IncHI1B.R27._1_R27, IncFIA.HI1._1_HI1 Col440I_1, ColRNAI_1
Rissen	1	19CS0417^*^	19CS0425		19CS0421 19CS0398		blaTEM.1, aadA1, aadA2, qnrS1, dfrA12, sul2, sul3, tetM, cmlA1, floR^*^/tetA	IncHI1A, IncHI1B, Col440II_1, ColRNAI_1 ^*^/Col440II_1, ColRNAI_1
	2			19CS0416			*blaTEM.1, aac(3)-IId, aadA2, mcr-1, tetA, tetM, floR, lnuF*	IncFIB.AP001918._1, IncFIC.FII._1, IncI2_1_Delta, Col.BS512._1, Col156_1, Col440II_1, ColRNAI_1
	3				19CS0420		*tetA*	Col440II_1, ColRNAI_1
	4			19CS0396 19CS0423	19CS0394		*tetA*	Col440II_1, ColRNAI_1
	5				19CS0422		*blaTEM.1, aadA1, aadA2, dfrA12, sul1, sul3, tetA, cmlA1*	IncY_1, Col440II_1, ColRNAI_1

*In gray are the isolates carrying the identical IncHI*.

* *Isolate belonging to a cluster, but it carries different resistance genes and/or plasmid replicons*.

The six IncHI1 replicons belonging to the same ST were found in the samples collected in the slaughterhouse (splitting place) and in the market (pork and cutting board). They belonged mostly to different clusters, and they were found in both serovars (Table 5). Although the difference in these plasmids lies in some of the ARGs and their MGEs, all carried multiple ARGs.

## Discussion

*Salmonella* species is a zoonotic pathogen of major importance due to the high number of human infections and bacterial isolates with an increasing antibiotic resistance (38). In Vietnam, the main reservoirs are food-producing animals (3) such as pigs, where *Salmonella* is widely disseminated along the pig value chain. In this study, 23 isolates from the pig value chain were analyzed. They belonged to two important serovars in Vietnam, i.e., *S*. Rissen (5) ST469, which is distributed around the world (39, 61), and *S*. Derby ST40 (4, 9), which is one of the most common STs found in pork and poultry (40).

In Vietnam, the prevalence of *Salmonella* with AMR is high in pork, other retail meat, and seafood products (12, 13). Most of them are resistant against tetracycline, trimethoprim–sulfamethoxazole, chloramphenicol, and ampicillin (5, 9, 12). This is comparable to our study, where high resistance was detected against ampicillin (73.9%) and trimethoprim and trimethoprim/sulfamethoxazole (both 56.5%, Table 2), but not against tigecycline (17.4%).

In this study, all sequenced isolates carried at least one ARG. These genes conferred resistance to 11 antibiotic classes. In other Vietnamese studies, genes against 8 (13) and 11 (41) antibiotic classes were also found. In this study, the most frequently detected ARGs were against tetracycline, β-lactam antibiotics, chloramphenicol, and trimethoprim–sulfamethoxazole (Table 3). This had been also detected in previous publications on Vietnamese pigs (5, 9, 12, 13, 42). Genes *tetA* and/or *tetM* were identified in all isolates (Table 3), such as in pigs, sampled in 2019 in the same country (13). The *bla*_TEM.1_ and/or *bla*_*LAP*_, genes were detected in 65.21% (Table 3) of the isolates, which were also identified in Vietnamese *Salmonella* strains identified in pigs (13) and humans (41). Almost all ARGs detected in those studies were previously observed in Vietnamese strains (13, 41). The exception was the detection of *fosA7.3*; this gene was found in 56.5% of the samples. This high prevalence was also observed recently in France (43) and in Germany (44). *FosA* was included recently in the Resfinder database, which determines the importance of keeping track of the updates of public databases (43). No ESBL- and AmpC-producing isolates were detected in our study, whereas in other published studies in Vietnam, such strains were also detected in pigs (5) and in humans (41). One isolate carried *mcr-1* (Table 3), which was also detected in Vietnamese pigs (8) and poultry samples (14).

Furthermore, MDR *Salmonella* species are also widely disseminated in Vietnamese pigs (5, 8), poultry (13), seafood (5), and humans (41). In our study, 69% of the strains were MDR, and *S*. Derby carried more ARGs than *S*. Rissen. This high multidrug resistance prevalence could be related to the use of large amounts of antimicrobials to treat infections, as well as to increase the productivity of animal farming (8). Making proper antimicrobial treatment using phenotypical and/or genotypical AMR detection (45) is one of the solutions for the reduction of the use of antimicrobials and for the dropping of resistance against first-line antimicrobials (46). For example, in this study, most *S*. Derby isolates were multiresistant (Table 1); thus, in the case of animal treatment, only the last-resort antibiotics, such as second- and third-generation cephalosporins, could be used. Nevertheless, these antibiotics are critically important for human treatment (47). *S*. Rissen isolates were mostly resistant against tetracycline (Table 1); a possible treatment for these animals would be florfenicol in feed, which is frequently used in Vietnam (48). However, other measures must be taken. For example, in Vietnam, there is a decree (13/2020/ND-CP) that sets the timeframe (between 2021 and 2026) for a ban on antimicrobials for prophylactic purposes in feed and animals, which was enacted in 2020 (49). In addition, the improvement of management practices and the use of vaccines and probiotics are the alternatives against growth-promoting and prophylactics (50).

In most isolates, there is a lack of correspondence between the detected phenotype and the genotype in at least one of the antibiotic-resistant families. This may be due to inadequate gene expression, which has been shown to be a barrier against drug resistance detection (51) or because the isolates have an MIC one dilution step above the ECOFF (52). In the case of aminoglycosides, only four strains were detected as resistant, and these strains presented from one up to three genes against aminoglycosides (Table 4). This could be related to the problem that *Salmonella* species does not show resistance against aminoglycosides *in vitro* (53). In the case of the tetracycline family, all the isolates presented at least one ARG (*tetA* and/or *tetM*); nevertheless, 14 strains were determined as wild type by the VITEK 2 Compact System. An explanation could be that the VITEK cards used in this study contain only tigecycline. The gene *tetA* decreases to some extent the sensitivity to tigecycline in *Salmonella* species (54), and *tetM* seems not to be a determinant in the resistance against tigecycline (13). The gene *fosA7* and *fosA7.3* have been previously detected in *S*. Derby in France, but no resistance *in vitro* has been tested (43). *S*. Heidelberg shows a high level of resistance when this gene was located in plasmids (55); however, in this study, all *S*. Derby isolates that carried *fosA7.3* were susceptible in both VITEK 2 Compact System (Table 4) and Micronaut AST-system (Supplementary Table 2). For this reason, further investigations must be done to validate the *fosA7.3* functionality in *S*. Derby. In conclusion, these discordances show that the genotypical determination of the AMR could add relevant information to the phenotypic detection.

Plasmids are usual vehicles for ARGs and can be spread by horizontal gene transfer even between broadly divergent bacteria (15, 56, 57). In this study, 95.6% (22/23) of the isolates carried at least one plasmid replicon. Col-plasmid replicons (Table 1) were detected in 91.3% (21/23) of the strains. These small mobilizable plasmids encode the bacteriocin colicin (15), and they have been frequently found in *Salmonella* species in the United States (58). However, plasmids of incompatibility groups IncI, IncFI, IncN, and IncHI carried the highest variety of ARGs in *Salmonella enterica* (16, 17). In this study, 34.7% (8/23) of the isolates carried at least one of these replicons (Table 1).

The IncHI plasmid is mostly found in *Salmonella* (15, 16, 59). This plasmid replicon was detected in 26.1% (6/23) of the samples. They belonged to the same MLST (unknown, nearest ST:16). Furthermore, plasmid p19CS0402-IncHI1 harbored an IncFIA replicon; this is a multireplicon MDR IncHI1/IncFIA type found previously in an *Escherichia coli* strain collected in China (60). This replicon was also found in strains 19CS0400, 19CS0401, and 19CS0403 (Tables 1, 5). Plasmids shared a common backbone (core genes and genes for replication, maintenance, and transmission), but the differences are MGE and ARGs, as described in previous studies (15, 16, 56).

Multiple ARGs are frequently carried by IncHI (16, 56). In this study, IncHI1 plasmid sequences carried between 3 and 10 ARGs. Genes *tetM* and *qnrS1* were present in all strains, but other genes, such as *folP2, qacE, ant1_2, cmlA1, ant1_3*, and *dfrA_12 bla*_TEM_, were also found in the plasmids_._ These MGEs, ARGs, and other accessory regions are acquired during the spread of the plasmid (16, 56), making IncHI larger than the other conjugative plasmids (15).

In this study, phylogenetic SNP analysis detected 10 clusters (Table 5). Five (three *S*. Derby and two *S*. Rissen) grouped together isolates of the slaughterhouse (splitting place, worker hands, and swab on pig carcass) and the market (pork and cutting board) (Table 5), suggesting a transmission of *Salmonella* from the slaughterhouse to the market. Furthermore, in three clusters (Table 5), *Salmonella* was found on the workers' hands and on the carcasses at the slaughterhouse. This suggests that cross-contamination of carcasses may happen by the handling of the workers or other procedures at the slaughterhouse (4). This shows the importance of sanitation and good hygienic procedures during slaughtering. These measures are necessary for the control and reduction of *Salmonella* transmission in pork meat (13).

Almost all clusters contained isolates with the same ARGs and plasmid replicons (Table 3). For example, *S*. Derby cluster 2 carried isolates with 13 ARGs and 2 replicons. These isolates were found in the slaughterhouse and on a cutting board at the market (Table 5), again suggesting the possible transfer of *Salmonella* and their resistance genes between the slaughterhouse and the market. This kind of transmission was detected in a previous study in Vietnam (12).

However, possible ARG transmission was not only seen within single clusters. Six isolates that belonged to serovars Rissen and Derby and belonged to four different clusters (based on SNP typing, Table 5, gray cells) carried an MDR IncHI1 plasmid with identical plasmid ST. These isolates were found in the slaughterhouse (splitting place) and at the market (pork and cutting board, Table 5, gray cells). Observations like this reveal the potential spread and distribution of a plasmid transmitting multidrug resistance in different clusters and serovars along the pig value chain.

Limitations to this study include the moderate number of strains obtained from samples from Vietnam. However, the data presented here highlight the importance of antibiotic resistance among *Salmonella* strains in Vietnamese pigs and show its potential spread to humans. Furthermore, a comparison of our strains with the same serovar and ST from pigs slaughtered in Vietnam was not possible because of the limited number of public sequences strain with the same characteristic as our strains. For this reason, our publication will add information to increase the number of sequence data to the public databases.

## Conclusions

Here, we report an analysis of 13 *S*. Derby and 10 *S*. Rissen isolates, collected in 2013 at various stages in pig slaughterhouses and pork markets in Vietnam. VITEK 2 Compact System determined AMR phenotype, and WGS was used for revealing ARGs, plasmids, and the phylogenetic relationships between the strains. The 86.9% (20/23) of the samples were resistant to at least one antibiotic, and all isolates carried at least one ARG, whereas 69.5% (16/23) of them were MDR. The most frequently detected ARGs conferred resistance against tetracycline, β-lactam antibiotics, chloramphenicol, and sulfonamide. One isolate carried *mcr-1*, but no ESBL- and AmpC-encoded genes were found in any of the isolates. However, the relation between the phenotype and the ARGs was discordant in the samples. Some of the ARGs were found on plasmids such as IncHI1, which accounted for 26.1% of the samples and carried between 3 and 10 ARGs. SNP analysis showed the potential of transmission of MDR *Salmonella* from slaughterhouse to market via the pork chain. Based on data from this study, ARGs, located in IncHI1 plasmids, could be a potential factor for the spread of multidrug resistance among clusters, serovars, and along the chain.

To investigate the MDR situation in the Vietnam pig value chain, further studies should be done using WGS to identify ARGs and track MDR strains and plasmids. In addition, the location of ARGs in plasmids or the chromosome could help to monitor and control the spread of ARGs. WGS and bioinformatics tools should be introduced as a standard procedure to help the identification of critical points within pork production chains to control the spread of antimicrobial-resistant *Salmonella*.

## Data Availability Statement

The datasets presented in this study can be found in online repositories. The names of the repository/repositories and accession number(s) can be found in the article//Supplementary Material.

## Ethics Statement

Ethical review and approval was not required for the animal study because Samples were collected from slaughterhouse and market environmental samples, pork meat and carcasses.

## Author Contributions

BG-S, RF, and HT contributed to conception and design of the study. BG-S performed the laboratory work and wrote the manuscript. BG-S and SG-S bioinformatic analysis. SD-X collected the samples. MA-G performed the minion sequencing. DM and RF provided the samples. All authors contributed to manuscript revision, read, and approved the submitted version.

## Conflict of Interest

The authors declare that the research was conducted in the absence of any commercial or financial relationships that could be construed as a potential conflict of interest.

## Publisher's Note

All claims expressed in this article are solely those of the authors and do not necessarily represent those of their affiliated organizations, or those of the publisher, the editors and the reviewers. Any product that may be evaluated in this article, or claim that may be made by its manufacturer, is not guaranteed or endorsed by the publisher.

## Supplementary Material

Supplementary Table 1 p19CS0402- IncHI1 plasmid annotation.

Supplementary Table 2 Micronaut AST for Fosfomycin in the 13 *S*. Derby isolates.

Supplementary Table 3 Single-nucleotide polymorphism (SNP) analysis.

Supplementary Table 4 IncHI1 plasmid isolates' identity percentage comparison.

Supplementary Table 5 Genome accession number NCBI.
